# Membrane lipidomics in schizophrenia patients: a correlational study with clinical and cognitive manifestations

**DOI:** 10.1038/tp.2016.142

**Published:** 2016-10-04

**Authors:** C Tessier, K Sweers, A Frajerman, H Bergaoui, F Ferreri, C Delva, N Lapidus, A Lamaziere, J P Roiser, M De Hert, P Nuss

**Affiliations:** 1 INSERM ERL 1157, CHU Saint-Antoine, Paris, France; 2 Service de psychiatrie et de psychologie médicale, Hôpital Saint-Antoine, AP-HP, UPMC Université Paris 06, Paris, France; 3 UPC KU Leuven, Kortenberg, Belgium; 4 SYLIA-STAT, Bourg-la-Reine, France; 5 Institut Pierre Louis d'épidémiologie et de Santé Publique, UMRS 1136, INSERM, Sorbonne Universités, UPMC Université Paris 06, Paris, France; 6 Public Health Department, Saint-Antoine Hospital, AP-HP, Paris, France; 7 UMR 7203, Laboratoire des biomolécules, Sorbonne Universités-UPMC Université Paris 06, Paris, France; 8 UCL Institute of Cognitive Neuroscience, London, UK

## Abstract

Schizophrenia is a severe mental condition in which several lipid abnormalities—either structural or metabolic—have been described. We tested the hypothesis that an abnormality in membrane lipid composition may contribute to aberrant dopamine signaling, and thereby symptoms and cognitive impairment, in schizophrenia (SCZ) patients. Antipsychotic-medicated and clinically stable SCZ outpatients (*n=*74) were compared with matched healthy subjects (HC, *n=*40). A lipidomic analysis was performed in red blood cell (RBC) membranes examining the major phospholipid (PL) classes and their associated fatty acids (FAs). Clinical manifestations were examined using the positive and negative syndrome scale (PANSS). Cognitive function was assessed using the Continuous Performance Test, Salience Attribution Test and Wisconsin Card Sorting Test. Sphingomyelin (SM) percentage was the lipid abnormality most robustly associated with a schizophrenia diagnosis. Two groups of patients were defined. The first group (SCZ c/SM−) is characterized by a low SM membrane content. In this group, all other PL classes, plasmalogen and key polyunsaturated FAs known to be involved in brain function, were significantly modified, identifying a very specific membrane lipid cluster. The second patient group (SCZ c/SM+) was similar to HCs in terms of RBC membrane SM composition. Compared with SCZ c/SM+, SCZ c/SM− patients were characterized by significantly more severe PANSS total, positive, disorganized/cognitive and excited psychopathology. Cognitive performance was also significantly poorer in this subgroup. These data show that a specific RBC membrane lipid cluster is associated with clinical and cognitive manifestations of dopamine dysfunction in schizophrenia patients. We speculate that this membrane lipid abnormality influences presynaptic dopamine signaling.

## Introduction

Schizophrenia is a chronic multifactorial disorder characterized by a number of symptom dimensions, cognitive abnormalities and functional impairment. These features are highly variable among individuals with schizophrenia, resulting in great heterogeneity in clinical presentation. Several explanatory models have been proposed to conceptualize the combination of these various characteristics. To date, none have accounted for the overall diversity of the observed manifestations, and most of the proposed models allow only a partial understanding of the disorder. The lipid hypothesis of schizophrenia of Horrobin^1^ was conceived in this framework, proposing to reconcile apparently heterogeneous schizophrenia features and also suggesting a biological basis for disease vulnerability.

Today, advances in lipidomics such as high-performance liquid chromatography, electrospray ionization and mass spectrometry have contributed to a more in-depth understanding of lipid physiology and allowed further developments of the original phospholipid (PL) hypothesis of schizophrenia. Lipids are now understood as versatile and dynamic regulators of numerous cellular processes that encompass, among others, signaling, budding and fusion of vesicles.^2^ Lipids are also known to move rapidly in the plane as well as across the bilayer in a dynamic and highly organized manner, particularly in red blood cell (RBC),^3^ to control various cellular activities.^4^ In this context, the lipid pattern abnormality in membranes is conceived as a trait or vulnerability marker associated with the disorder.

The new field of neurolipidomics seeks to understand how dynamic changes in membrane composition regulate brain cell function. The lipid composition of brain membranes is brain-region specific,^5^ but is also governed by mechanisms that control the membrane composition in cells throughout the body. Brain signaling may thus be assessed indirectly via the study of the lipid composition of membrane in peripheral cells such as RBCs.^6, 7, 8^ Membrane abnormalities in schizophrenia patients were demonstrated via measurement of the membrane PL ratio,^9, 10^ turnover^11^ and inner/outer distribution,^12^ as well as quantification and identification of the polyunsaturated fatty acids (PUFAs) from PL.^13^

Here, we undertook a lipidomic study of the RBC membrane of chronic medicated schizophrenia patients to identify abnormal membrane lipid clusters associated with the disorder, and also to distinguish patient subgroups. On the basis of studies in lipid-deprived animals showing that abnormal lipid metabolism is associated with disrupted dopamine dysfunction,^14^ we sought to examine whether specific membrane lipid clusters are associated with dopamine-related symptomatology and cognition in patients with schizophrenia.

## Materials and methods

### Study population

The study included 74 antipsychotic-medicated and clinically stable outpatients with schizophrenia (SCZ, DSM-IV-TR criteria) and not meeting criteria for another treated DSM-IV axis III disorder. The healthy control (HC) group was composed of 40 subjects matched for age and education level, recruited among hospital staff and students with no personal and family history of psychosis and/or bipolar disorder. In both the groups, exclusion criteria included cholesterol-lowering treatments and dietary supplementation with PUFAs. The metabolic syndrome was not an exclusion criterion, except when it required a pharmacological treatment following a clinician's opinion. Cholesterol-lowering medication was an exclusion criterion because membrane and circulating cholesterol can exchange to some points and cannot be considered independent compartments in terms of cholesterol content. Food intake was assessed in patients through a dietary questionnaire.^15^ The patients and controls provided written informed consent in a protocol approved by the Medical Ethics Committee of the University Psychiatric Centre (EC/UC/(2011-16), Katholieke Universiteit Leuven, campus Kortenberg (Belgium)). The demographic and clinical characteristics of the study samples are summarized in Table 1. Antipsychotic medication consisted of either typical (haloperidol, chlorpromazine, flupentixol) or atypical compounds (amisulpride, aripiprazole, clozapine, olanzapine, quetiapine, sertindole and risperidone).

### Clinical and cognitive measures

Patients' psychopathology, general functioning and cognition were assessed by a trained nurse. The Positive and Negative Syndrome Scale (PANSS),^16^ Global Assessment of Functioning and Clinical Global Impression scales have been completed. The cognitive tasks were administrated using a computerized platform. Three tests have been used: AX Continuous Performance Test (CPT-AX), Salience Attribution Test (SAT) and Wisconsin Card Sorting Test (WCST).

The CPT-AX is a measure of sustained attention and working memory, consisting of a series of trials on which a single letter is presented briefly in the middle of the screen. This version of the test also included different colored (that is, visually salient) stimuli on 20% of the trials, adding a perceptual salience component to the test.^17^ The task was programmed in PXLAB.^18^

The SAT is a test of reward learning and motivational responding, on which participants must respond to a target stimulus immediately preceded by a cue, which signals the probability of gaining a reward, the magnitude of which is higher for quicker responses.^19^ The task was programmed in Cogent, a stimulus presentation toolbox for Matlab.

The WCST is a test evaluating mental flexibility and working memory,^20^ on which participants must sort cards according to different rules, which must be learned from feedback and change periodically. We used the WCST Research Edition Version 2 (http://www.parinc.com).

### Biological measures

The lipid composition of the RBC membrane was studied to identify and measure all major lipid classes: phosphatidylcholine (PC), phosphatidylserine (PS), sphingomyelin (SM), phosphatidylethanolamine (PE), PE plasmalogen and their molecular species. The number of the major molecular species studied was 45, 18, 23 and 42 for PC, PS, SM and PE, respectively. PE was further studied as a function of its location in the outer and inner membrane leaflet. Both diacyl phosphatidylethanolamine (DPE) and monoacyl phosphatidylethanolamine (LPE), which together comprise PE, were measured on each of the membrane leaflet.

The detailed presentation of the blood sample preparation and data acquisition of phospholipids with liquid chromatography-tandem mass spectromerty (LC-MS/MS) for complete lipid data analysis is provided in the Supplementary Methods (Lipid measures). In brief, total lipids were extracted from the RBC cell membranes based on the methods of Folch *et al.*^21^ Outer leaflet and total membrane PE labeling was made using trinitro-benzylsulfonic acid (Sigma-Aldrich, Saint-Quentin Fallavier, France). Separation of PL classes were obtained using high-performance liquid chromatography (HPLC) solvent gradient program and spectrometry identification parameters (Supplementary Tables S-A to D, Supplementary Methods). The application of HPLC solvent gradient and mass spectrometer scan functions were controlled by the Analyst Software (AB Sciex, Les Ulis, France) data system. The samples were analyzed using an electrospray ionization tandem mass spectrometry (ESI/MS/MS, API3000, TQ, Applied Biosystems-Sciex, Concord, ON, Canada) either with scan mode or multiple-reaction monitoring. A comprehensive description of the methodology can be found in Brugger *et al.*^22^ Multiple-reaction monitoring was used to measure the distribution of DPE and LPE between the two RBC membrane leaflets. The complete lipid data were acquired using Analyst 4.2.2 software. To identify and quantify spectral peaks, LIMSA software was used.^23^

### Statistical data analysis

The data were analyzed using SAS 9.3. The groups were compared using *t*-tests for continuous variables or a non-parametric Mann–Whitney Wilcoxon test when parametric assumptions were violated (normality tested with the Kolmogorov–Smirnov test). Categorical data were analyzed using chi-square tests. Correlations with RBC PLs were investigated using Spearman's correlation tests. A step-wise logistic multivariate analysis including PS, SM, PC, PE, external DPE, external LPE and PE plasmalogen was performed to determine which lipid variables were most reliably associated with schizophrenia diagnosis. All the tests were two-sided with a statistical significance level set at *P=*0.05. For cognitive and clinical data one-way and two-way analyses of variance with SM group and diagnosis group as fixed factors and the SM × diagnosis interaction term were estimated. Box-Cox power transformation of continuous variables was used to limit departures from normality assumptions. Results are given with the F_k_ statistic including the number of degrees of freedom, k. Multimodality of the SM distribution was tested to assess whether the distribution was a mixture of normal laws, using a clustering analysis with bayesian information criterion for information criterion.^24^

## Results

As shown in Table 1, the SCZ and HC groups did not differ in terms of age, sex and education level.

### Membrane lipid composition differs in patients and healthy controls

As shown in Table 2a, a univariate analysis of the RBC lipids identified significantly lower SM percentage in the SCZ group compared with the HC group (27.38 vs 30.59%), along with a concomitant higher PS percentage (8.73 vs 6.64%).

### Decreased membrane sphingomyelin percentage allowed distinguish four study populations

In a multiple logistic regression analysis using membrane PL values to predict schizophrenia diagnosis, only SM was selected (odds-ratio estimate of 0.833 with 95% Wald Confidence Limits (0.744–0.933), *P=*0.0003), confirming its strong association with diagnosis. The SM percentage appeared to follow a ‘bimodal' distribution, that is a mixture of two normal laws with means 23.2 and 22.5% and same variance 5.4. A threshold value of SM percentage was then determined to classify individuals according to mean SM percentage value: (1) those in the range of HCs; and (2) those below this value. A receiver operating characteristic curve identified an SM cutoff of 28.58 (mean %) to maximize the Youden index. Only 22.5% of the HCs exhibited an ‘abnormal' SM percentage while this was identified in 55.4% of the schizophrenia patients (chi-square Q=10.12, *P=*0.0015).

**Figure 1:**
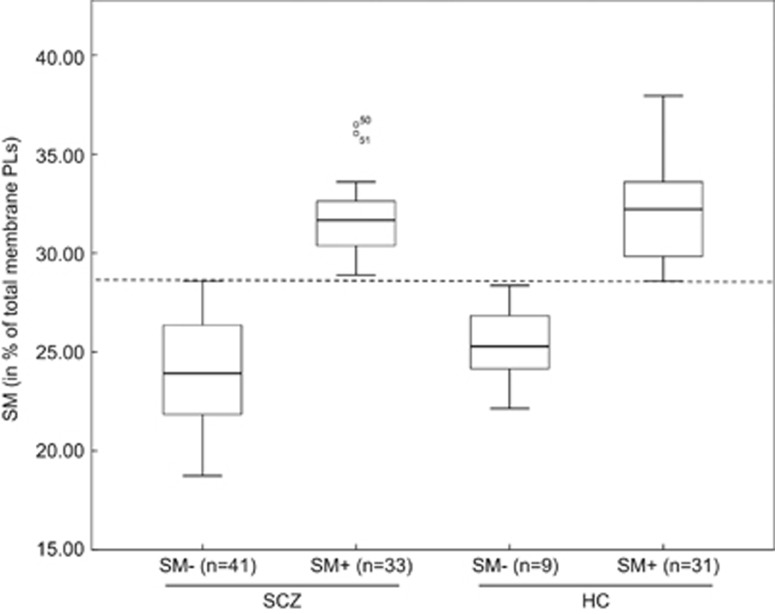
Distribution of the schizophrenia (SCZ) and healthy control (HC) samples as a function of their mean SM percentage content in the RBC membrane. PL, phospholipid; RBC, red blood cell; SM, sphingomyelin.

Two clusters of membrane lipid constitutions can be described, identifying two population groups. The group named ‘cluster/SM−' (c/SM−) is constituted of individuals whose RBC membrane comprises a SM mean percentage below 28.58. By contrast, the c/SM+ group has a mean SM percentage above 28.58 (in the range of the majority of HCs). Four populations can thus be distinguished among the study participants: SCZ c/SM− (*n=*41), SCZ c/SM+ (*n=*33), HC c/SM− (*n=*9) and HC c/SM+ (*n=*31). These four groups are represented on Figure 1.

For biophysical and biological reasons, an isolated decrease of a membrane PL is not possible without complex and compensatory changes in the ratio of the other membrane containing lipids. Thus, SM status cannot be conceived of in isolation, but considered as a marker of a broader membrane lipid dysfunction. Compared with their c/SM+ counterparts, each of the c/SM− patient and HC subgroups exhibit a different cluster of compensatory lipids (Table 2b).

### Membrane lipid composition and distribution differs between c/SM+ and c/SM− patient populations

As mentioned above, in the c/SM− patient subgroup, significant concomitant decreases in PC and PE plasmalogen, along with significant increases in PS and PE percentages, were observed (Table 2b). These results indicate a very different RBC membrane lipid composition in the c/SM− and c/SM+ patients. The PE percentages were further characterized as a function of the location of PE in the membrane leaflet (inner- vs outer-located PE). Compared with the c/SM+ subgroup, c/SM− patients also differed in DPE/LPE distribution. The c/SM− subgroup exhibited a significant increase in DPE (both external and internal) and a decrease in LPE (both external and internal; Table 2b).

In addition, key PUFAs known to be modified in membranes in schizophrenia patients were compared between the c/SM− and c/SM+ patients. The molecular species comprising either one or two of the following PUFAs: linoleic acid (C18:2 n-6), arachidonic acid (C20:4 n-6), docosapentaenoic acid (C22:5, n-3) and docosahexaenoic acid (C22:6 n-3) were examined. A significant difference in these molecular species percentage was observed between the two patient subgroups (Supplementary Table S1, Supplementary Results). This difference was most pronounced for the PE molecular species. The PUFA molecular species profile for LPE and DPE also differed significantly in terms of their outer/inner membrane leaflet distribution.

Importantly, the c/SM+ and c/SM− SCZ subgroups did not differ in age, gender, education level, age of onset, type or disease duration (Table 4), or amount of antipsychotic medication (calculated using the chlorpromazine equivalence measure from Danivas *et al.*^25^).

### The membrane lipid cluster is not identical in c/SM− patients and controls

The compensatory lipid changes associated with the low SM subgroups are population-specific with the c/SM− patients deprived in PE and enriched in PS relative to the c/SM− HCs. Further, the molecular species also differed among the c/SM− SCZ/HC subgroups in terms of total membrane content and asymmetrical distribution (Supplementary Table S2, Supplementary Results).

### Cognitive measurements

As shown in Table 3, compared with the number of subjects for which lipid and clinical data were available, the number of individuals able to perform the cognitive tasks was lower and variable as a function of the specific test, thus reducing the statistical power of these comparisons. Despite this, highly significant results are observed for some cognitive tasks as a function of SM status.

### CPT-AX test

The mean scores for hit rate and reaction time differed significantly between the SCZ and HC groups (Supplementary Table S3, Supplementary Results). As shown in Table 3, among the SCZ patients, the c/SM− subjects had shorter mean reaction times and higher false alarm scores relative to the c/SM+ subjects. However, the interactions between SM status and diagnostic group were nonsignificant for both of these variables.

### SAT (first block data only)

The scores for mean response time and explicit adaptive salience differed significantly between the SCZ and HC groups (Supplementary Table S3, Supplementary Results). Among the SCZ patients, the c/SM− subjects had lower explicit adaptive salience scores relative to the c/SM+ subjects (Table 3), and the interaction between SM status and diagnostic group was significant.

Notably, only half (*n=*12/26) of the c/SM− SCZ patients that completed the first SAT block were able to continue to the second block, whereas all of the c/SM+ subgroup (*n=*21) completed both blocks, indicating a poorer capacity to be involved in extended cognitive testing for the c/SM− patient subgroup.

### WCST test

The mean scores for trials, errors, perseverative responses, perseverative errors and non-perseverative errors were significantly higher (worse) in SCZ compared with HC (Supplementary Table S3, Supplementary Results).

Among the SCZ patients, the c/SM− subjects had poorer performance with significantly more trials, errors, perseverative responses, perseverative errors and non-perseverative errors (Table 3). Interestingly, SM status was also associated with trials, errors and non-perseverative errors in the HC group, but in the opposite direction, with more errors in the c/SM+ group. There were significant interactions between SM status and diagnostic group for all these variables.

### Summary

On all the three cognitive tests, the SCZ patients performed worse overall than the HCs, but this difference was largely driven by the c/SM− subgroup of patients. However, among the HCs, SM status was not associated with impaired cognitive performance. Supplementary Table S4 (Supplementary Results) shows that most of the cognitive scores were worse in the c/SM− patients compared with the c/SM− HC, whereas the c/SM+ HC and SCZ subgroups scored similarly on many of the variables (data presented in Table 3, analyses not shown).

### Clinical measures

The PANSS scale scores were compared between the c/SM− and c/SM+ patients (Table 4). PANSS total, positive and general psychopathology scores were significantly higher in the c/SM− compared with the c/SM+ subgroup. The c/SM− patients scored also significantly higher on the positive, disorganized/cognitive and excited subscales of the five dimensional segmentation of the PANSS, with a trend on the anxiety/depression subscale.^26^ No differences were observed between the c/SM subgroups for the PANSS negative subscale. Of interest, there were twice as many severe patients (assessed by a PANSS total cutoff of 45) in the c/SM− group compared with the c/SM+ group.

The more severe WCST impairment in the c/SM− sample is unlikely to be explained by higher PANSS scores: after adjusting for the PANSS total score (Supplementary Table S5, Supplementary Results), SM status remained associated with trials, errors, perseverative responses, perseverative errors and non-perseverative errors.

## Discussion

Consistent with the existing literature, the present study demonstrated that the RBC membrane PL composition differs between stabilized medicated schizophrenia individuals and matched HCs. Two membrane lipid clusters that aggregate with either low (c/SM−) or normal (c/SM+) membrane SM content allowed the identification of two groups of patients distinguishable by the ratio of all membrane PLs, inner/outer PE distribution and fatty-acid composition. Compared with the c/SM+ patients, the c/SM− SCZ subjects are characterized by significantly more severe psychopathology and impaired cognitive performance.

### Lipid data

As far as we know, our study is first in identifying the four most important PL classes using LC-MS/MS. In addition, while in other studies, the total fatty-acid content was measured irrespective of their PL origin, here the PL molecular species could be identified in an individualized basis for each of them. The pattern of membrane PL alteration in schizophrenia noted in previous studies is comparable with the data presented here. Significantly lower quantities of PE and PC,^27^ as well as SM^28^ were found in post-mortem brain tissues from patients with schizophrenia. In the present study, an overall increase of PE was observed. However, our results showing a significant decrease in RBC membrane mean SM percentage are in agreement with another post-mortem study showing a decreased SM and increased PS.^28^ In particular, the decreased SM percentage observed in the c/SM− patients is consistent with data showing a myelin sheath alteration in schizophrenia as evidenced by imaging and post-mortem studies.^29, 30^

Like many other lipids, SM participates in signaling via its transmutation into bioactive molecules or indirectly by biophysical changes in the synapse. At the plasma membrane level, the concentration of SM can be regulated by multiple enzymes. In particular, a sphingomyelinase/sphingomyelin synthase system is involved that controls the membrane levels of both SM and ceramide. These lipids can also be metabolized to other bioactive sphingolipids.^31^ SM is a key PL involved in membrane microdomain formation via its aggregation with cholesterol allowing membrane lipid partition.^32^ These SM-enriched domains, called rafts, are involved in the compartmentalization of cellular processes and also serve as platforms for many cellular signaling activities^33^ in which budding and fusion of vesicles are involved.^34^

The total membrane FA content in schizophrenia patients has also been investigated previously. In particular, Bentsen *et al.*^35^ were able to demonstrate a very highly significant bimodal distribution of PUFA among patients, mirroring the present results but on total membrane PUFAs. In line with most prior results,^13^ our study identified significant differences in PUFA content in the molecular species of PC, PS and PE known to be key for PUFA content.^36^ Compared with the c/SM+ subgroup, the c/SM− patients significantly differed in the content of PUFA molecular species. The specific inner/outer distribution and FA composition of DPE and LPE observed for each of the SM groups is in accordance with the assumption that the membrane homeostasis of these two populations differs not only in composition but also distribution. This also confirms our previous finding showing an abnormal inner/outer distribution of PE in a subgroup of schizophrenia patients.^12^

A dysfunctional plasmalogen dynamic in the plasma and platelets of schizophrenia patients has been described for the PE plasmalogens^37^ and was interpreted as an indirect marker of increased oxidative stress in these patients.^38^ In line with these results, we identified a significant decrease in PE plasmalogen in the c/SM− subgroup, which was also characterized by more severe clinical and cognitive features. In this subgroup, the plasmalogen decrease was associated with a PE increase. This modification is in agreement with a plasmalogen-deprived animal model, in which a compensatory increase in PE was described, which presumably occurs to maintain the total membrane amount of PE/PE plasmalogens.^39^

Several human studies in schizophrenia and animal models^40^ have emphasized the role of antipsychotic medication in relation to the membrane PL abnormalities observed in schizophrenia patients. A correcting effect of antipsychotic compounds on the membrane lipid composition has been described^41^ involving several mechanisms.^42, 43^ In the present study, all patients were chronically treated by antipsychotics with no differences in between SM subgroups in terms of typical/atypical ratio and medication daily dosage. The difference of membrane lipid composition observed between the SM subgroups is thus unlikely to be related to the type or dose of the prescribed antipsychotic treatment.

### Membrane lipid composition, psychopathology and cognitive performance

#### PANSS scores

Total, positive, disorganized/cognitive as well as excited PANSS scores were significantly higher in the c/SM− compared with the c/SM+ patients. Such an effect of PL composition in the RBC membrane has been previously examined in few studies taking into account the PL^12^ and PUFA^44^ content in the RBC membrane of patients. Low concentrations of arachidonic acid and docosahexaenoic acid have been described in patients with predominantly negative symptoms.^45, 46^ In our study, no differences in the negative subscale scores were observed between the SM subgroups.

#### WCST test

In the present study, the membrane lipid content has been examined in relation to cognitive performance and compared with data from ultra-high risk individuals,^47^ as well as treated and stabilized patients.^48^ The observed findings in the c/SM− patient subgroup, potentially indicate a more severe impairment of prefrontal cortex function relative to the c/SM+ patient subgroup. Interestingly, a reverse pattern was observed in HCs, with SM− status being associated with better performance relative to c/SM+ (Table 3). Animal studies of chronic deficiency in n-3 PUFA support the hypothesis that cognitive impairment associated with PL/FA membrane changes is caused by a more active mesolimbic dopamine pathway and a less active mesocortical pathway.^49, 50^ In line with this finding, we found that the c/SM− patients, who exhibited a more pronounced PUFA dysfunction, manifested both more positive symptoms and WCST deficits relative to the c/SM+ patients.

### SAT test

The dopamine dysregulation described in patients with schizophrenia is thought to be reflected in aberrant reward processing.^51^ In the present study, SAT scores differed between the SM patient subgroups, with the c/SM− patients having lower explicit adaptive salience. Consistent with our results, a study aiming to examine the extent to which some psychotic symptoms reflect aberrant salience, Roiser *et al.*^19^ demonstrated that patients with first episode psychosis exhibited reduced explicit adaptive salience relative to controls, which has been replicated independently.^52^ The results presented here suggest that PL abnormalities may contribute to this disrupted reward learning. However, implicit aberrant salience scores were not different between the SM patient subgroups in the present study.

### CPT-AX test

Intact prefrontal processing is needed to perform the CPT-AX test.^53, 54^ In the present study, surprisingly, the c/SM− patients had shorter reaction times compared with the c/SM+ patients. One interpretation is that in the c/SM− patients, a more potent effect of the salient stimuli enabled them to perform the task better compared with the c/SM+ subgroup. Another explanation is that the c/SM− group responded faster because they were less concerned about making errors, in other words adopting a more impulsive response style (a speed-accuracy trade-off). This latter interpretation is supported by a concomitant increase in the number of false alarms committed compared with the c/SM+ patients, also indicative of poorer prefrontal function. That said, it should be noted that the diagnostic group-by-SM status interactions were nonsignificant for these variables, which lessens support for these interpretations

### More pronounced dopamine dysfunction in c/SM− schizophrenia individuals?

The differences between patients and HCs on all the three cognitive tests were largely driven by the c/SM− subgroup. In addition, c/SM− status was associated with more severe PANSS total and positive scores. We speculate that these results have some connection with dopamine dysfunction in c/SM− relative to c/SM+ patients. Indeed more severe positive symptoms,^55^ disorganization^56^ and cognitive impairment^57, 58^ have been shown to be associated with dopamine dysregulation. Our data also consistent with animal models of chronic dietary n-3 PUFA deficiency^59^ suggest that the c/SM− patients exhibit more severe dopamine dysfunction in both the striatum and the prefrontal cortex compared with the c/SM+ individuals.

## Conclusion

We found evidence that a specific RBC membrane lipid cluster was associated with differences in dopamine-related clinical and cognitive manifestations in schizophrenia patients. No other studied clinical or treatment variables could account for these differences. We hypothesize that the difference in clinical and cognitive manifestations between the two SM patient groups can, in part, be explained by differences in presynaptic dopamine release as converging evidence indicates such dysfunction in schizophrenia.^60, 61, 62^

Nonetheless, this study does have some limitations. In particular, fewer patients underwent the cognitive tests compared with the number of those included in the membrane lipid study, and the cognitive tests we used are only indirectly related to dopamine dysfunction. A direct measure of dopamine release would provide more convincing evidence that membrane lipid content affects dopamine bioavailability in schizophrenia patients. In addition, our finding cannot be generalized to acute and/or low functioning patients. Furthermore, general conditions that participate in the lipid bioavailability such as gut malabsorption, in particular inflammatory bowel status, biliary acid turnover, and non-celiac and celiac gluten sensitivity have not been addressed in the present study.

Structural lipid biomarkers are a rapidly evolving field, in particular for neuropsychiatric disorders where even subtle perturbations in the lipid content of neurons and myelin can disrupt their function. Considerable further investigation is needed, including the validation of these findings in other patient populations with brain imaging to appraise dopamine dysfunction. Further questions such as the therapeutic effect of a membrane lipid content-adjusted PUFA supplementation or the identification of genetic and morphologic differences between various membrane lipid endophenotypes also represent exciting areas for further research.

## Figures and Tables

**Table 1:** Demographic and clinical data

Characteristics	*SCZ*		*HC*		*Chi-square* Q	*MWW* Z	P*-value*
	*Mean*	*s.d.*	*Mean*	*s.d.*			
*N*	74		40				
Gender: male (% of total population)	48 (64.8%)		24 (60%)		0.747 (1 d.o.f.)		0.387
Age (years)	43.8	9.3	42.6	13.2		0.462	0.644
*Education level (in % of total)*					3.329 (3 d.o.f.)		0.344
Lower High School	3 (4.1%)		1 (2.5%)				
High School	39 (53.4%)		15 (37.5%)				
College	17 (23.3%)		12 (30.0%)				
University	14 (19.2%)		12 (30.0%)				
Age of onset (year)	24.6	6.2	—				
Duration of illness (years)	22.3	6.4	—				
Number of hospitalizations	7.8	5.5	—				
Chlorpromazine equivalent (mg)	480.2	383.4	—				
CGI (mean)	3.03	0.74	—				
GAF (mean)	66.7	8.5	—				
PANSS (mean total score)	48.5	16.3	—				

Abbreviations: CGI, Clinical Global Impression; d.o.f., degree of freedom; GAF, Global Assessment of Functioning; HC, healthy control; MWW, Mann–Whitney Wilcoxon test; PANSS, Positive and Negative Syndrome Scale; SCZ, schizophrenia.

Comparisons were performed with chi-square tests for categorical data and Mann–Whitney Wilcoxon tests for quantitative data.

**Table 2:** Phospholipid data from schizophrenia patients and healthy controls

*a*	*Total population*	*SCZ (*n=*74)*		*HC (*n=*40)*		*MWW* Z	P*-value*
	*Phospholipid*	*Mean (%)*	*s.d.*	*Mean (%)*	*s.d.*		
	PE	22.40	7.15	19.81	8.38	1.59	0.112
	PC	41.49	6.03	42.96	6.06	1.12	0.263
	SM	27.38	4.51	30.59	3.76	3.35	**0.001**
	PS	8.73	3.31	6.64	2.12	3.33	**0.001**

*b*		SCZ (n=74)				P*-value*	HC (n=40)				P*-value*
	*Subgroups*	*c/SM− (*n=*41)*		*c/SM+ (*n=*33)*			*c/SM− (*n=*9)*		*c/SM+ (*n=*31)*		
	*Phospholipid*	*Mean (%)*	*s.d.*	*Mean (%)*	*s.d.*		*Mean (%)*	*s.d.*	*Mean (%)*	*s.d.*	
	PE	27.12	4.23	16.53	5.48	**<0.0001**	28.61	8.86	31.51	5.74	0.2570
	PC	38.64	5.07	45.04	5.24	**<0.0001**	36.27	3.63	44.90	5.18	**0.0003**
	SM	23.92	2.69	31.66	1.76	**<0.0001**	23.92	2.69	31.66	1.76	**<0.0001**
	PS	10.31	3.44	6.77	1.72	**<0.0001**	7.42	2.40	6.42	2.01	0.2065
	Plasmalogen PE	5.34	0.76	6.53	0.17	**<0.0001**	5.76	0.72	6.89	1.30	**0.0121**
											
	*Outer/Inner leaflet PE*										
	Outer PE (DPE+LPE)	6.62	2.17	6.57	2.28	0.9653	6.12	1.81	6.88	3.04	0.7955
	DPE	4.94	1.36	4.18	1.32	**0.0120**	4.25	1.31	4.16	1.67	0.4761
	LPE	1.68	1.47	2.39	1.20	**0.0007**	1.87	0.81	2.73	1.51	0.1450
	Inner PE (DPE+LPE)	26.40	5.11	27.84	5.20	0.1919	24.30	5.74	26.38	6.82	0.4563
	DPE	22.16	5.17	19.18	4.24	**0.0322**	19.57	5.35	20.43	5.67	0.9226
	LPE	4.24	2.55	8.66	4.18	**<0.0001**	4.72	1.64	5.95	2.16	0.1363

Abbreviations: DPE, diacyl phosphatidylethanolamine; LPE, monoacyl phosphatidylethanolamine; PC phosphatidylcholine; PE, phosphatidylethanolamine; PS, phosphatidylserine; SM, sphingomyelin.

The section (a) shows the mean ratio for the major phospholipid (PL) classes from RBC membranes in schizophrenia patients (SCZ) and healthy controls (HC). Section (b) indicates the PL mean percentage in schizophrenia and healthy controls for each SM subgroup. *P*-values are derived from the non-parametric Mann–Whitney Wilcoxon (MWW *Z*) test. Significant findings are in bold.

**Table 3:** Cognitive data from schizophrenia and healthy control SM subgroups

	*SCZ*				*F1*	P*-value*	*HC*				*F1*	P*-value*
	*c/SM−*		*c/SM+*				*c/SM−*		*c/SM+*			
	*Mean*	*s.d.*	*Mean*	*s.d.*			*Mean*	*s.d.*	*Mean*	*s.d.*		
*CPT-AX*	*(*n=*36)*		*(*n=*28)*				*(*n=*7)*		*(*n=*29)*			
Hit rate	85.71	23.41	78.57	28.24	0.729	0.396	98.98	2.7	95.32	7.7	1.115	0.299
Reaction time mean	498.58	101.4	584.41	167.54	4.943	**0.030**	444.3	62.5	469.45	79.78	1.138	0.294
False alarm	9.08	12.86	6.73	13.85	4.59	**0.036**	5.49	3.06	5.36	8.63	0.653	0.425
								
*SAT1*	*(*n=*26)*		*(*n=*21)*				*(*n=*7)*		*(*n=*26)*			
Response time mean	350.8	88.3	349.43	94.89	0.01	0.922	313.61	29.5	299.23	58.45	1.32	0.259
Imp Adap Sal	8.83	42.63	1.83	58.55	0.224	0.638	28.58	20.12	13.11	32.82	1.393	0.247
Imp Aber Sal	31.17	22.56	22.06	21.19	2.787	0.102	29.71	20.82	22.31	17.86	1.573	0.455
Exp Adap Sal	1.25	28.86	28.33	31.79	10.02	**0.003**	46.07	28.68	35.1	29.03	0.798	0.379
Exp Aber Sal	6.83	7.47	7.86	14.02	1.758	0.192	8.21	7.03	6.63	8.48	0.487	0.490
Money	853.77	329.93	792.05	364.02	0.466	0.498	1004.7	287.22	909.19	313.7	0.546	0.466
								
*WCST*	*(*n=*22)*		*(*n=*12)*				*(*n=*5)*		*(*n=*19)*			
Trials	106.45	23.09	89.58	21.25	4.242	**0.048**	72.8	6.98	86.68	17.11	4.644	**0.042**
Correct responses	64.05	16.02	69.42	9.14	0.685	0.414	65.2	3.96	70.11	6.53	2.358	0.139
Errors	42.41	31.28	20.17	19.05	5.636	**0.024**	7.6	3.29	16.58	11.12	8.648	**0.007**
Pers responses	23.95	21.7	12.08	16.02	6.781	**0.014**	4.4	1.67	9.21	7.82	3.604	0.071
Pers errors	21	17.55	11.08	13.71	6.524	**0.016**	4.4	1.67	8.47	6.83	3.588	0.071
Non pers errors	21.41	18.39	9.08	6.08	4.86	**0.035**	3.2	2.17	8.11	5.32	11.28	**0.003**

Abbreviations: CPT-AX, Continuous Performance Task AX; Exp aber sal, explicit aberrant salience; Exp adap sal, explicit adaptive salience; HC, healthy control; Imp aber sal, implicit aberrant salience; Imp adap sal, implicit adaptive salience; Non pers errors, non-perseverative errors; Pers errors, perseverative errors; Pers responses, perseverative response; SAT, salience attribution test; SCZ, schizophrenia; SM, sphingomyelin; WCST, Wisconsin Card Sorting Test.

*P*-values are derived from one-way analysis of variance model on power-transformed data. Significant findings are in bold.

**Table 4:** Psychopathology scores in both schizophrenia SM subgroups

	*SCZ*				P*-value*
	*c/SM− (*n=*41)*		*c/SM+ (*n=*33)*		
	*Mean*	*s.d.*	*Mean*	*s.d.*	
*PANSS*					
Total	53.32	18.45	42.42	10.78	**0.010**
Positive	11.61	4.86	9.00	2.88	**0.014**
Negative	14.71	6.60	12.36	4.84	0.149
General	27.00	9.72	21.06	5.68	**0.008**
					
*PANSS (5 factors)*					
Positive	10.41	5.09	7.48	2.09	**0.009**
Negative	16.76	8.18	13.36	5.08	0.151
Cognitive/disorganized	11.44	4.05	9.52	2.56	**0.041**
Excited	5.20	2.42	4.30	0.81	**0.038**
Anxiety/depression	9.51	3.70	7.76	2.61	0.060
					
CGI	3.10	0.83	2.94	0.61	0.292
GAF	66.10	9.32	67.42	7.36	0.675
Age (years)	43.39	8.34	44.31	10.47	0.334
Gender: male (% of total population)	28 (70.0%)		22 (66.7%)		1
Age of onset (year)	24.59	5.75	24.64	6.89	0.838
					
*Education level (%)*					0.397
Lower High School	2 (5.0%)		1 (3.0%)		
High School	24 (60.0%)		15 (45.4%)		
College	9 (22.5%)		8 (24.2%)		
University	5 (22.4%)		9 (27.3%)		
Chlorpromazine equivalent (mg)	464.6	350	529.5	426	0.474
Disease duration (years)	18.18	6.82	20.34	8.95	0.448

Abbreviations: CGI, Clinical Global Impression; GAF, Global Assessment of Functioning; PANSS, Positive and Negative Syndrome Scale; SCZ, schizophrenia; SM, sphingomyelin.

*P*-values are derived from one-way analysis of variance model the non-parametric Mann–Whitney Wilcoxon (MWW *Z*) test.

Significant findings are in bold.
