# Supplementary material for: Membrane lipidomics in schizophrenia patients: a correlational study with clinical and cognitive manifestations
Source: Transl Psychiatry. 2016 Oct 4;6(10):e906–. doi: 10.1038/tp.2016.142 (PMC5315538; doi:10.1038/tp.2016.142)
Supplement: Supplementary Tables [file tp2016142x1.doc]

**Supplemental Results**

**Table S1.**

Significant differences in key PUFAs molecular species in schizophrenia SM sub-groups

P-values derived from the non-parametric Mann-Whitney Wilcoxon test.

Abbreviations: PE, phosphatidylethanolamine; PC phosphatidylcholine; PS, phosphatidylserine; LPE, monoacyl phosphatidylethanolamine. PExx:y: xx=sum of carbon acyl chains and y=number of unsaturation; C18:2, Linoleic acid; C20:4, Arachidonic acid, C22:5, Docosapentaenoic acid; and C22:6, Docosahexaenoic acid are the polyunsaturated fatty acids of interest.

**Table S2**

Membrane phospholipid and molecular species composition comparison between c/SM- patients and healthy controls in total membrane and outer/inner leaflet for the PE species.

In c/SM- individuals, the PL membrane composition is not identical in patients and HC. Compared to healthy controls, the membrane of patients is significantly deprived in PE and enriched in PS. Other significant molecular species differences are also observed between both c/SM- populations

**Table S3.**

Comparison of the CPT-AX, SAT, and WCST values between schizophrenia and healthy control individuals

P-values derived from one-way analysis of variance model on power-transformed data.

Abbreviations: CPT-AX, Continuous Performance Task AX; SAT, Salience attribution test; WCST, Wisconsin Card Sorting Test.

**Table S4.**

Comparison of the CPT-AX, SAT, and WCST values between schizophrenia and healthy control individuals in c/SM- sub-groups

P-values derived from one-way analysis of variance model on power-transformed data.

Abbreviations: CPT-AX, Continuous Performance Task AX; SAT, Salience attribution test; WCST, Wisconsin Card Sorting Test.

**Table S5.**

Comparison of the CPT-AX, SAT, and WCST values in c/SM- and c/SM+ patients adjusted for the PANSS Total scores

P-values derived from one-way analysis of variance model on power-transformed data.

Abbreviations: CPT-AX, Continuous Performance Task AX; SAT, Salience attribution test; WCST, Wisconsin Card Sorting Test. 
