# Supplementary material for: Membrane lipidomics in schizophrenia patients: a correlational study with clinical and cognitive manifestations
Source: Transl Psychiatry. 2016 Oct 4;6(10):e906–. doi: 10.1038/tp.2016.142 (PMC5315538; doi:10.1038/tp.2016.142)
Supplement: Supplementary Information [file tp2016142x2.doc]

**SUPPLEMENTAL METHODS**

**Lipid measures**

*Blood sample*

A fasting blood sample was taken from both patients and HC. Samples were collected in citric acid dextrose tubes via venepuncture at the tertiary-care psychiatric unit at the Kortenberg University Hospital (Leuwen, Belgium). The blood samples were stored at 4°C and transferred within three days to the Mass Spectroscopy Department at the Saint-Antoine Hospital (tertiary care University Hospital, Paris, France) where lipid extraction, PL and FA identification and quantification were performed.

*Sample preparation for mass spectrometry analysis*

RBC membranes were washed three times with a buffer consisting of 150 mM NaCl and 20 mM Tris HCl (pH 7.7) and centrifuged (10 min, 3000 rpm). Total lipids were extracted from the RBC cell membranes based on the methods of Folch et al.

*Outer and total membrane PE labeling*. Freshly-washed RBCs (250 µl) were incubated with 30 µl (9 mM) of trinitro-benzylsulfonic acid (TNBS, Sigma-Aldrich, France) under non-permeant conditions at 5°C for 30 min in 250µl of 150 mM NaCl and 20mM Tris HCl buffer supplemented with NaHCO3 (40µl, 1mM). The reaction was stopped by the addition of 30 µL 0.5 M HCl. PE quantification was made possible by adding 1 µg of internal standard consisting of diacyl PE (C17:0 C20:4) (Avanti polar lipids, France) also labeled with TNBS. After evaporation under azote flux, 1 ml of chloroform/methanol (C/M 8/2) was added. The sample was divided in two equal volumes. The first volume (external PETNB) was evaporated under N2 flux and then 1 mL of C/M (10/90) was added under argon and stored at -20°C until analysis. The second (total PETNB) was used for the total PE labelling. After evaporation of the solvent under N2 flux, 400 µl of physiological serum with 5 µl de TNBS (5% w/v) and 40 µl of NaHCO3 at 1 mM concentration were added. Incubation at room temperature (1 h) was performed and then the aqueous solution was removed under speed vacuum. A volume of 1 mL of C/M (10/90) was added under argon and stored at -20°C until analysis. External PETNB and total PETNB samples were finally prepared by a dilution of 2.5 and 25 respectively in the HPLC solvent.

*Data acquisition of phospholipids with LC-MS/MS*

Lipid extracts were suspended in 200 µl cyclohexane/isopropanol/water/ ammonium acetate 500 mM (58/40/0/2) volume (Solvent A). For phospholipid analysis, a total of 10 µL was injected onto a 3.0 mm × 250 mm length PVA-SIL column (YMC Europe GmbH, D-46514, Schermbeck, Germany), at a flow rate of 150 µL/min, with a total run time of 70 min. A 2 mm frit cap and a short reverse phase guard cartridge (in-line guard C18-silica, 3 µm, 4 X 20 mm2, CIL-Cluzeau, 92419, Courbevoie, France) were used to prevent the capillary clogging. A passage through the guard cartridge was used to decrease ion suppression. The mobile phase gradient used consisted of solvent A and solvent B (cyclohexane/isopropanol/water/ammonium acetate 500 mM (50/40/8/2). In each measurement, gradient elution was applied to separate each lipid classes; the detailed gradient program and other LC conditions are listed below (Tables S-A to S-D). The application of HPLC solvent gradient and mass spectrometer scan functions were controlled by the Analyst© Software (AB Sciex) data system. The samples were analyzed using an electrospray ionization tandem mass spectrometry (ESI/MS/MS, API3000, TQ, Applied Biosystems-Sciex, Concord, Ontario, Canada) either with scan mode or multiple-reaction monitoring (MRM). The specific detection of lipid classes was based on the mass-to-charge-ratio (m/z) value of their precursor ion scanning, which was related to their head group fragments. The scans were made in negative ion mode. Based on the precursor ion scanning value, the PL was identified at 184 (m/z) for PC and SM, 185 (m/z) for PS and neutral loss scanning 141 (m/z) for PE. A comprehensive description of the methodology can be found in Brugger et al. MRM was used to measure the distribution of DPE and LPE between the two RBC membrane leaflets.

*Complete lipid data analysis*

The data were acquired using Analyst 4.2.2 software. To identify and quantify spectral peaks, LIMSA software was used. This tool is capable of finding and integrating peaks in a mass spectrum, matching the peaks with a user-supplied list of expected lipids, correcting for overlap in their isotopic patterns, and finally identifying the corresponding molecular species. In this study, each m/z peak was confirmed using the online Lipidmaps mass spectrometry peak prediction data set ([http://www.lipidmaps.org](http://www.lipidmaps.org/)). The quantitative data are provided in percentages.

**Table S-A**

**Liquid chromatography parameters used for phospholipid separation**


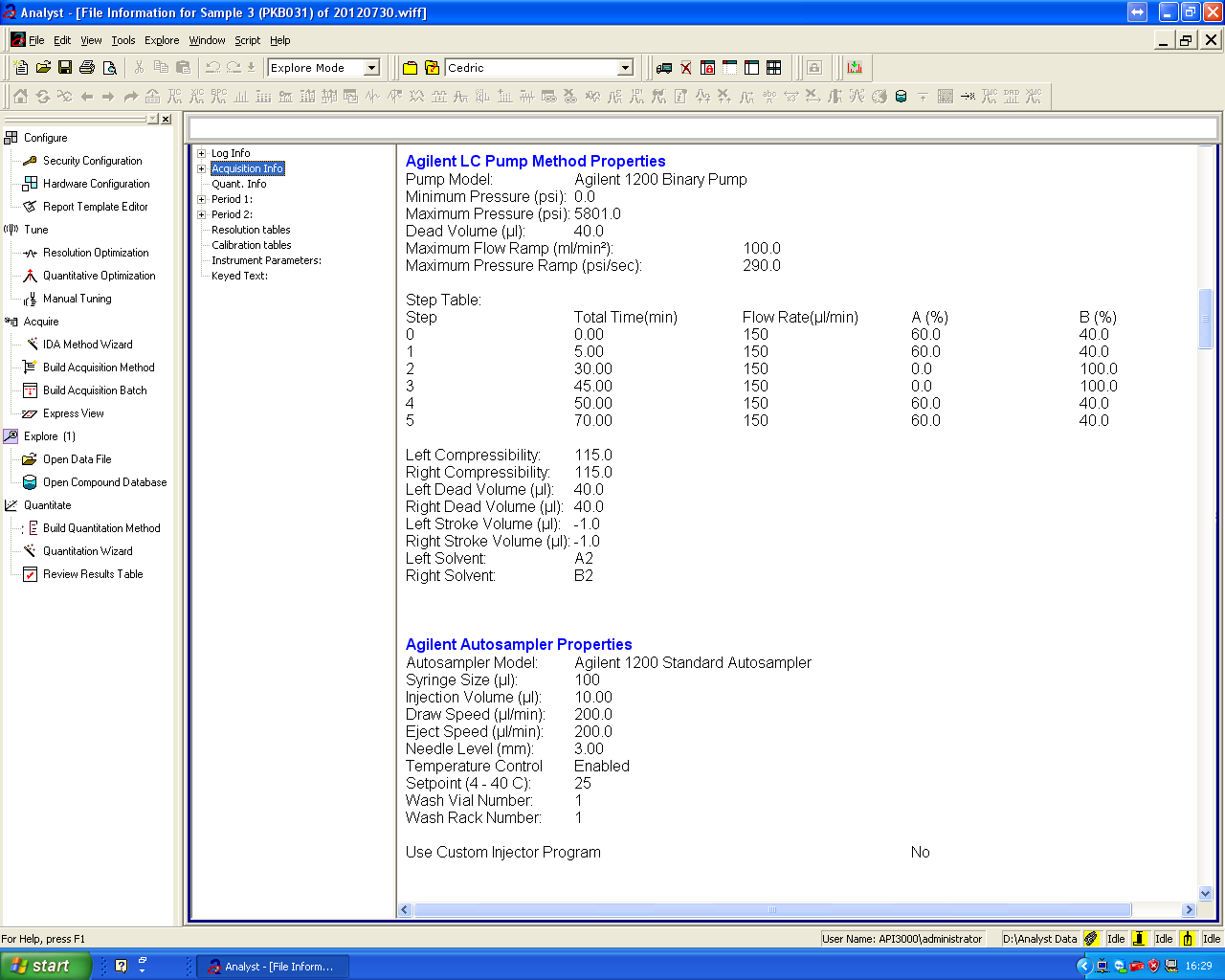


**Table S-B**

**Mass spectrometry parameters used for the analysis of phosphatidylethanolamine** (for period 1 in experiment 1), phosphatidylcholine/sphingomyelin (for period 2, in experiment 1) and phosphatidylserine (for period 2, in experiment 3)


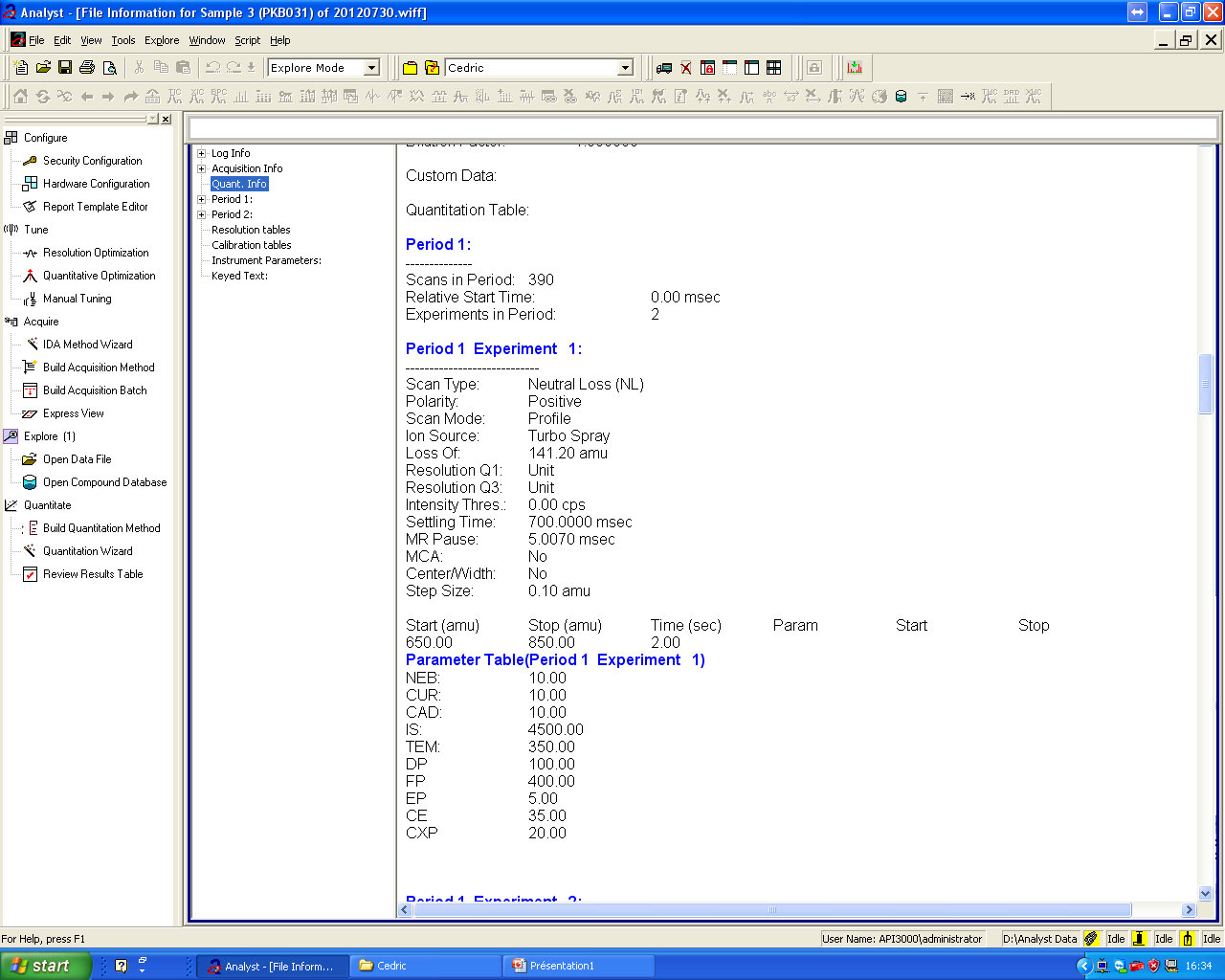


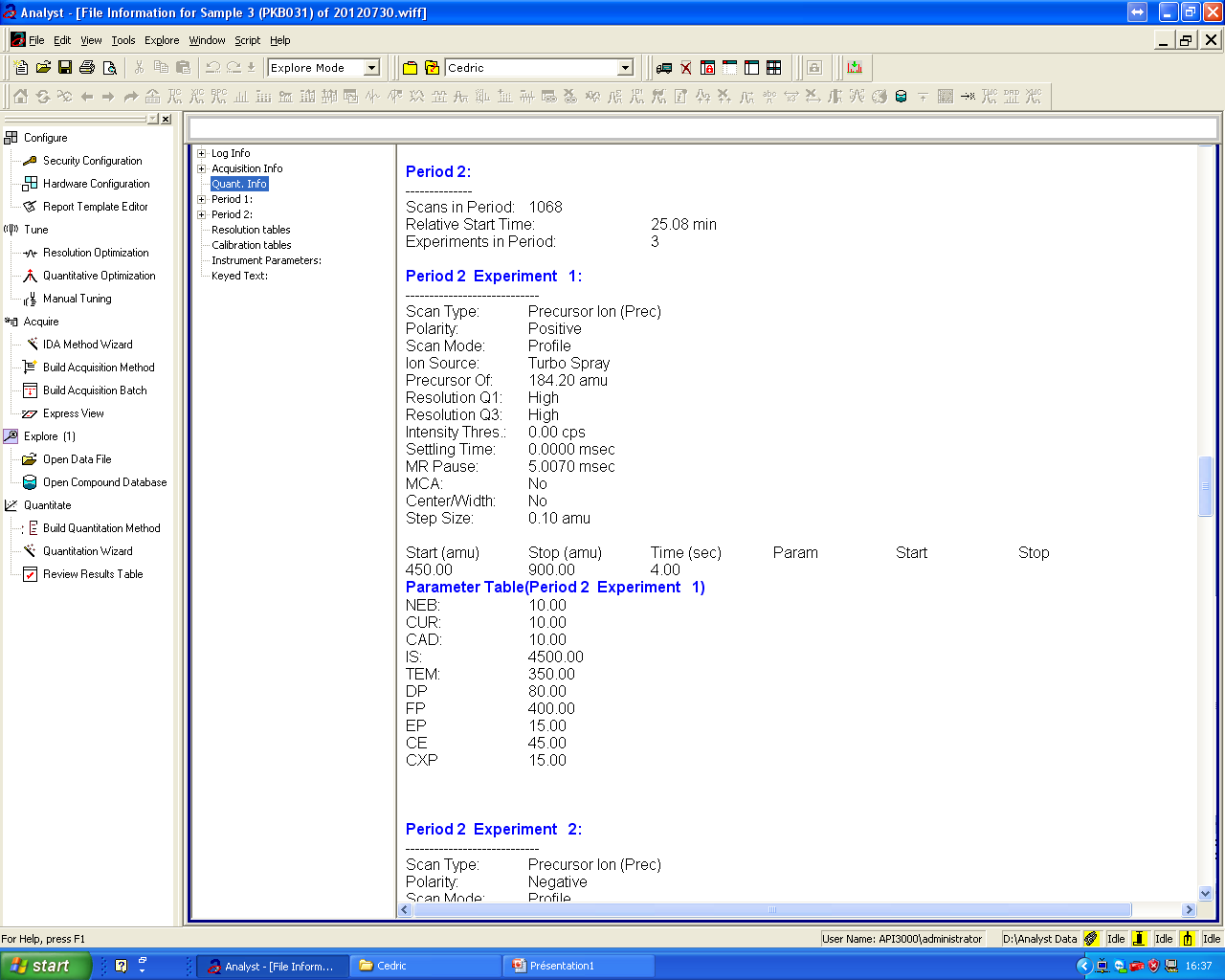


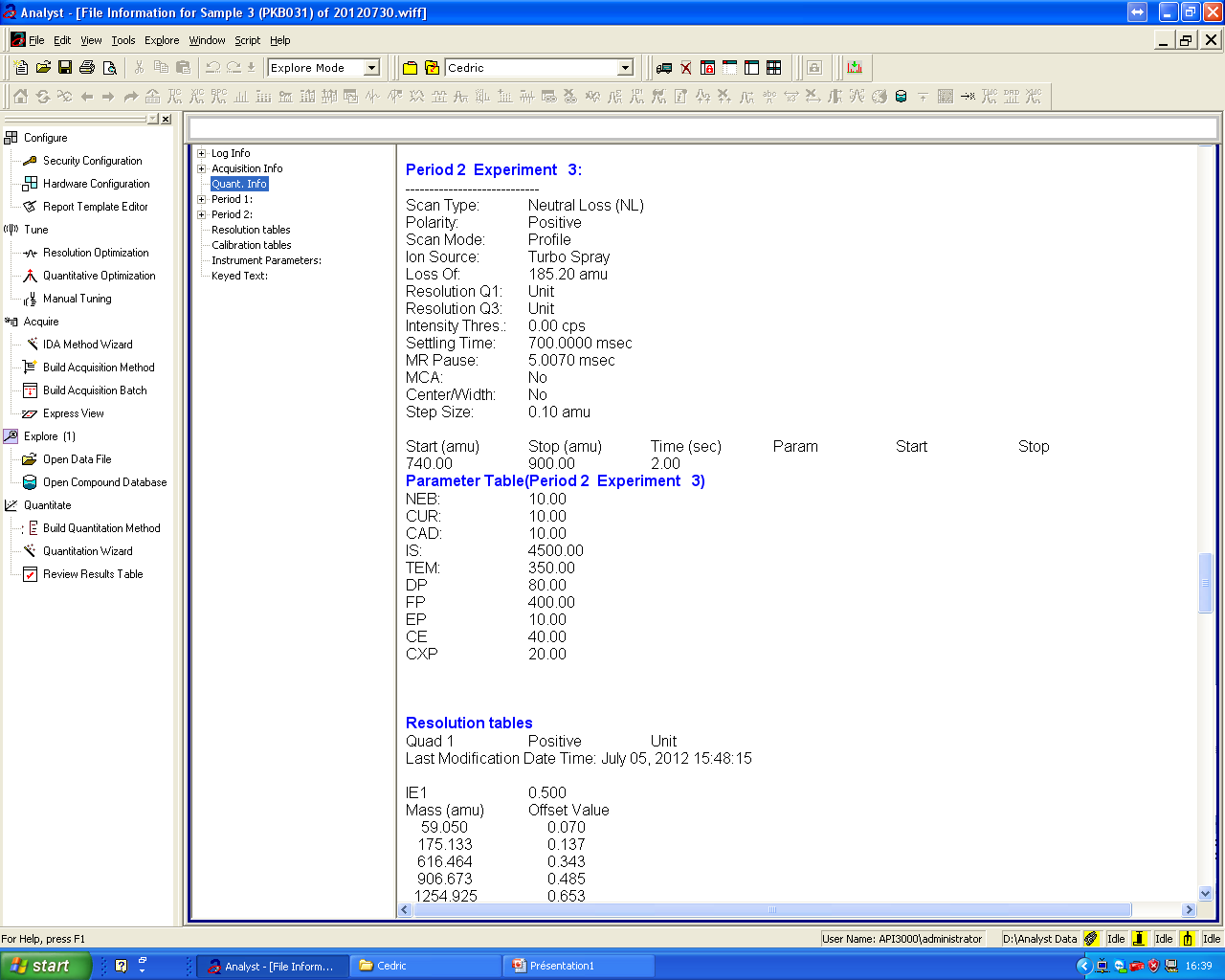


**Table S-C**

**Liquid chromatography parameters used for phosphatidylethanolamine-TNB analysis**


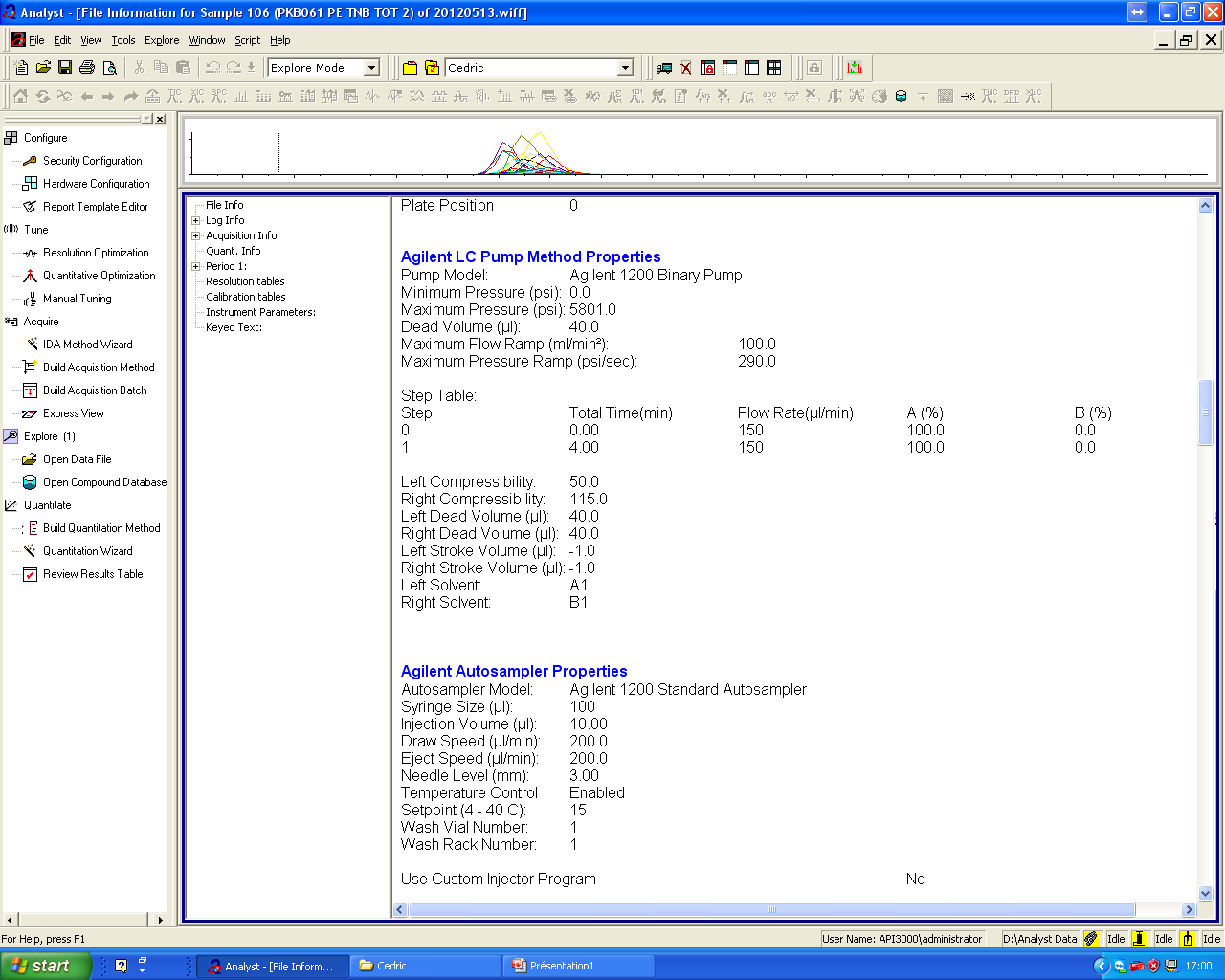


**Table S-D**

**Mass spectrometry parameters used for the phosphatidylethanolamine-TNB analysis and the list of Multiple Reaction Monitoring (MRM) for mass detection (Q1/Q3 (amu))**
